# Toll-like Receptor 4 Mediates the Inflammatory Responses and Matrix Protein Remodeling in Remote Non-Ischemic Myocardium in a Mouse Model of Myocardial Ischemia and Reperfusion

**DOI:** 10.1371/journal.pone.0121853

**Published:** 2015-03-30

**Authors:** Yufeng Zhai, Lihua Ao, Joseph C. Cleveland, Qingchun Zeng, T. Brett Reece, David A. Fullerton, Xianzhong Meng

**Affiliations:** 1 Department of Surgery, University of Colorado Denver, Aurora, Colorado, United States of America; 2 Department of Cardiology, Nanfang Hospital, Southern Medical University, Guangzhou, China; Emory University, UNITED STATES

## Abstract

The signaling mechanism that mediates inflammatory responses in remote non-ischemic myocardium following regional ischemia/reperfusion (I/R) remains incompletely understood. Myocardial Toll-like receptor 4 (TLR4) can be activated by multiple proteins released from injured cells and plays a role in myocardial inflammation and injury expansion. We tested the hypothesis that TLR4 occupies an important role in mediating the inflammatory responses and matrix protein remodeling in the remote non-ischemic myocardium following regional I/R injury. Methods and results: TLR4-defective (C3H/HeJ) and TLR4-competent (C3H/HeN) mice were subjected to coronary artery ligation (30 min) and reperfusion for 1, 3, 7 or 14 days. In TLR4-competent mice, levels of monocyte chemoattractant protein -1 (MCP-1), keratinocyte chemoattractant (KC), intercellular adhesion molecule 1 (ICAM-1) and vascular cell adhesion molecule 1 (VCAM-1) were elevated in the remote non-ischemic myocardium at day 1, 3, and 7 of reperfusion. Levels of collagen I, collagen IV, matrix metalloproteinase (MMP) 2 and MMP 9 were increased in the remote non-ischemic myocardium at day 7 and 14 of reperfusion. MMP 2 and MMP 9 activities were also increased. TLR4 deficiency resulted in a moderate reduction in myocardial infarct size. However, it markedly downgraded the changes in the levels of chemokines, adhesion molecules and matrix proteins in the remote non-ischemic myocardium. Further, left ventricular function at day 14 was significantly improved in TLR4-defective mice. In conclusion, TLR4 mediates the inflammatory responses and matrix protein remodeling in the remote non-ischemic myocardium following regional myocardial I/R injury and contributes to the mechanism of adverse cardiac remodeling.

## Introduction

Ischemic heart disease remains the major cause of morbidity and mortality. Myocardial inflammatory responses initiated by ischemia and reperfusion (I/R) worsen myocardial injury and matrix protein remodeling, which cause adverse cardiac remodeling and exaggerated heart failure following I/R injury [1].

Toll-like receptor 4 (TLR4) has been found to play a role in myocardial I/R injury in both regional and global I/R models [2–4]. Activation of this innate immunoreceptor by a variety of endogenous agents, termed as danger-associated molecular patterns (DAMPs), leads to the activation of pro-inflammatory signaling cascades. It is well known that pro-inflammatory signaling mediated by TLR4 involves tumor necrosis factor (TNF) receptor-associated factor 6 (TRAF6), interleukin (IL)-1 receptor-associated kinases (IRAKs), nuclear factor-kappaB (NF-κB)-inducing kinase (NIK), and the IκB kinase (IKK) [5–7]. IKKs degrades IκB, leading to the activation of NF-κB, a master pro-inflammatory transcription factor. Several studies show that TLR4-defective mice have reduced infarct size and attenuated myocardial overall inflammation following I/R [2], [8]. Our previous work on a global myocardial I/R model linked an improved functional recovery in TLR4-deifcient hearts to attenuated NF-κB activation and reduced cytokine production [4]. Further, we and others have demonstrated that myocardial tissue TLR4, rather than TLR4 in infiltrated leukocytes, has a critical role in mediating myocardial I/R injury [9], [10]. While the role of TLR4 in myocardial I/R injury following a short term of reperfusion has been extensively evaluated [2], [3], [11], few studies have determined the role of TLR4 in myocardial I/R injury and cardiac function in a prolonged time course.

Left ventricle (LV) remodeling following myocardial infarction is the reparative process triggered by an increase in work load to the uninjured myocardium, resulting in profound alterations of LV architecture with a discrete collagen scar, ventricular dilation and fibrosis in non-infarcted myocardium [12]. During the remodeling process, activated myocardial fibroblasts express extracellular matrix (ECM) proteins. It is known that ECM protein expression and matrix structure remodeling mainly occur in non-ischemic myocardium and in salvaged myocardium in the ischemic zone [13]. The overall inflammatory responses in the heart, including the remote non-ischemic myocardium, may play an important role in the ECM protein remodeling, which contributes to adverse cardiac remodeling and heart failure. However, the signaling mechanism that mediates the inflammatory responses and ECM protein remodeling in the remote non-ischemic myocardium remains unclear. It is likely that myocardial TLR4 signaling elicits the inflammatory responses in non-ischemic myocardium that in turn cause the dys-regulation of the remodeling process in non-ischemic myocardium.

We hypothesize that TLR4 occupies an important role in mediating the inflammatory responses and ECM protein remodeling in the remote non-ischemic myocardium and that elimination of TLR4-mediated inflammatory responses improves cardiac function following a long term of reperfusion. The purposes of the present study were to determine: 1) the effect of TLR4 deficiency on the expression of chemokines and adhesion molecules, as well as mononuclear cell accumulation in the myocardium, particularly in non-ischemic myocardium, in a murine model of regional myocardial I/R injury, 2) the effect of TLR4 deficiency on the expression of ECM proteins in non-ischemic myocardium, and 3) whether elimination of TLR4-mediated myocardial inflammatory responses improves cardiac function following prolonged reperfusion.

## Materials and Methods

### Animals

Male TLR4-competent (C3H/HeN) mice and male TLR4-defective (C3H/HeJ) mice, 16–20 weeks old and body weight 26–32 g, were used. TLR4-defective mice have a point mutation in the intracellular domain of TLR4, resulting in a complete loss of the signaling function of this innate immunoreceptor. Animals were acclimated in a quarantine room for 2 weeks before use.

The present study was carried out in strict accordance with the recommendations in the Guide for the Care and Use of Laboratory Animals of the National Institutes of Health. The protocol was approved by the Committee on the Ethics of Animal Experiments of the University of Colorado Denver [Permit Number: B-40513(09)2E]. Open-chest surgery was performed under ketamine and xylazine anesthesia, and all efforts were made to minimize suffering.

### Experimental protocol

Coronary artery ligation was performed to induce regional myocardial I/R. All surgical procedures were performed following the protocol described previously [14]. Briefly, mice were anesthetized with ketamine (60 mg/kg, ip) and xylazine (12 mg/kg, ip). Anesthetized animals were ventilated via tracheal intubation using a Harvard rodent respirator. Their body temperature was maintained at 36.8–37.2°C using a thermoregulated surgical table. The chest was opened by a left lateral thoracotomy, and a reversible coronary artery snare occluder was placed around the left descending coronary artery. The distance from the ligation site to the lower edge of the left auricle was approximately 2.0–3.0 mm. Evans blue dye staining confirmed consistent size of ischemic area. Animals were subjected to 30 min of ischemia followed by reperfusion by removing the knot and were observed for 1, 3, 7 or 14 days. In sham-operated mice, the suture was passed around the left descending coronary artery, but not tied. Sham animals were observed for 1, 3, 7 and 14 day to match the duration of I/R. Data from sham animals were combined since sham operation did not cause any temporal changes in the parameters examined.

### Pressure-volume hemodynamic analysis

LV pressure-volume analysis was performed in mice subjected to 14 days of reperfusion. All surgical procedures were performed following the protocol described previously [15]. Briefly, mice were anesthetized with pentobarbital sodium (Vortech Pharmaceuticals, Dearborn, MI; 50 mg/kg, ip) and anticoagulated with heparin (Elkins-Sinn, Cherry Hill, NJ; 1,000 units/kg, ip). Pentobarbital sodium was chosen for anesthesia since it provides good anesthesia without affecting heart rate. Therefore, cardiac function data can be obtained from hearts in with unaltered heart rate.

Animals were kept on a heating blanket, and core body temperature was maintained at 37°C ±0.5°C. A microcatheter (Millar Instruments, Houston, TX; 1.0 F) was inserted into LV through the right common carotid artery. Pressure-volume loops were recorded for 20 min using the MPVS-400 System with the aid of PVAN software (Millar Instruments, Houston, TX). Developed pressure, end-systolic volume, end-diastolic volume, ejection fraction and cardiac output were analyzed. For the assessment of maximal elastance, the abdomen was opened by right sub costal incision to reach the inferior vena cava. Caval occlusion was produced over a 3-second period using a nonmetallic occluder. At the end of the experiment, hearts were removed, and myocardium specimens from ischemic and non-ischemic (LV tissue above the occlusion site) zones were prepared.

#### Chemokine assay

Myocardial tissue was homogenized as described previously [9]. Levels of MCP-1 and KC were analyzed using enzyme-linked immunosorbent assay kits (R & D Systems, Minneapolis, MN) following the manufacturer’s protocol.

#### Fluorescence-based enzyme activity assay

Matrix metalloproteinase (MMP) 2 and MMP 9 activity was analyzed using fluorescence-based activity assay kits (Enzium, Philadelphia, PA) following the manufacturer’s instruction.

### Immunoblotting

Immunoblotting was applied to analyze adhesion molecules, MMPs and collagens in ischemic and non-ischemic myocardium. Myocardial homogenate was mixed with sample buffer (100 mM Tris-HCl, pH6.8, 2% SDS, 0.02% bromophenol blue and 10% glycerol). Crude protein (40 μg) was fractioned on 4–20% gradient acrylamide gels and transferred onto a nitrocellulose membrane (Bio-Rad Laboratories, Hercules, CA). Membranes were incubated in phosphate-buffered saline (PBS) containing 5% non-fat dry milk for 60 minutes to block non-specific binding. Then, membranes were probed with primary antibodies against ICAM-1, VCAM-1, MMP 2, MMP 9, TIMP-2, TIMP-1, collagen I or collagen IV. After washing with TPBS (PBS containing 0.05% Tween 20), the membranes were incubated with a peroxidase-linked secondary antibody specific to the primary antibody applied. Following further washes, membranes were incubated with enhanced chemiluminescence reagents. The membranes were then exposed on X-ray films. HSP 25, a constitutive small heat shock protein, was used for loading control in immunoblotting. NIH Image J software was used to analyze the area and density of protein bands.

### Histology and immunofluorescence staining

Myocardial tissue embedded in Tissue-Tek* CRYO-OCT compound was cryosectioned. Transverse tissue sections (5 μm in thickness) were stained with hematoxylin and eosin staining (HE) for histologically analysis of infarct size.

Immunofluorescence staining was performed to detect mononuclear cells in the myocardium. Tissue sections were fixed in 4% paraformaldehyde, incubated with a rat monoclonal antibody against mouse CD68^+^ (Serotec, Raleigh, NC), a specific marker protein for monocytes and macrophages, overnight at 4°C. After washing with PBS, sections were incubated with Cy3-tagged donkey polyclonal antibody against rat IgG (labeling monocytes and macrophages in red). Nuclei were stained with bis-benzimide (DAPI, labeling nuclei in blue), and cells were counterstained with Alexa 488-tagged wheat germ agglutinin (outlying cells in green). Microscopy was performed with a Leica DMRXA digital microscope (Leica Mikroskopie and Systeme GmbH, Wetzlar, Germany) equipped with Slidebook software (I. I. I., Denver, CO).

### Statistical analysis

Data are expressed as mean ± standard error (SEM). Statistical analysis was performed using StatView software (Abacus Concepts, Calabasas, CA). Student’s t-test was used for comparison between two groups. ANOVA with post hoc Bonferroni/Dunn test was performed to analyze differences with multiple group comparison. A difference was considered significant if *P* value < 0.05. Nonparametric Mann-Whitney U test was performed to confirm the difference of 2 group comparison. For multiple group comparison, nonparametric Kruskal-Wallis test was performed to confirm the difference.

## Results

### TLR4-defective mice exhibited smaller infarct sizes

**Fig 1:**
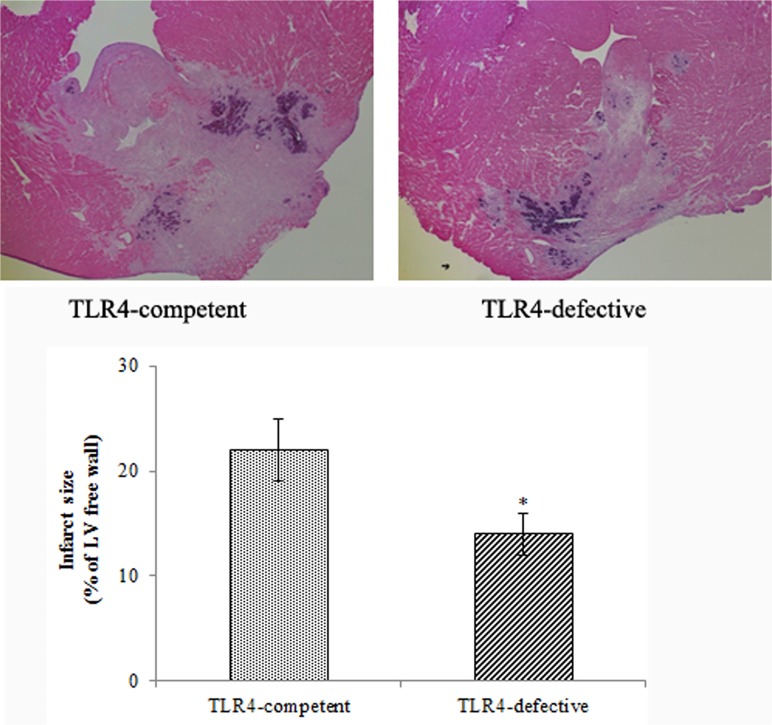
TLR4-defective mice display smaller infarct size at day 7 of reperfusion. Myocardial infarct size as a percentage of LV free wall was assessed by HE staining. Representative images show that infarct size is smaller in TLR4-defective mice than in TLR4-competent mice. Data are expressed as mean ± SE. n = 7 in each group; *P<0.05 vs. TLR4-competent.

HE staining was applied to heart sections after 7 days of reperfusion. TLR4-defective hearts had smaller infarct sizes (14 ± 1.1% of LV free wall, *P*<0.05) in comparison to TLR4-competent hearts (22 ± 2.1% of LV free wall) (Fig. 1).

### TLR4 deficiency attenuated the inflammatory responses in both ischemic and non-ischemic myocardium

**Fig 2:**
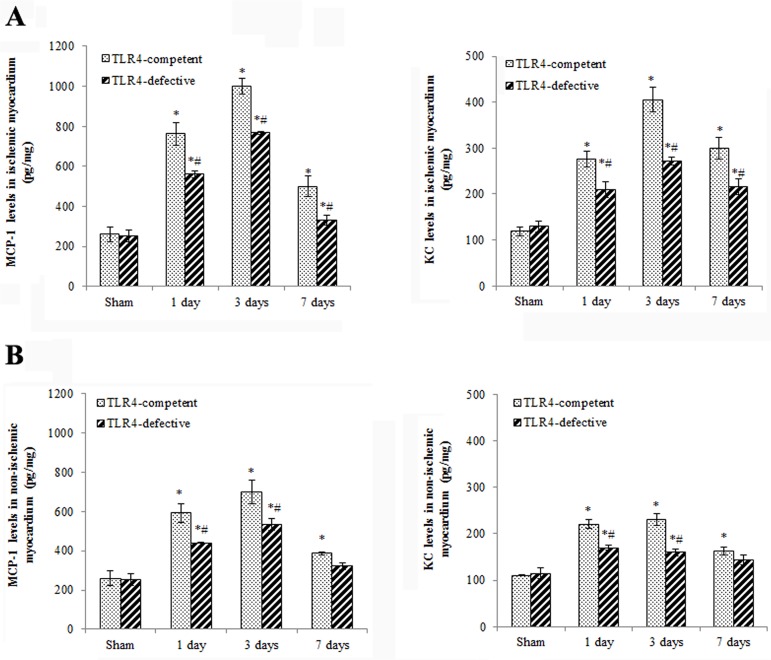
TLR4 deficiency reduces the expression of chemokines in ischemic and non-ischemic myocardium. Myocardial levels of MCP-1 and KC were analyzed at day 1, 3 and 7 of reperfusion. Lower chemokine levels were found in both ischemic (A) and non-ischemic (B) myocardium in TLR4-defective mice. Data are expressed as mean ± SE, n = 7 in each group, *P<0.05 vs. combined sham controls (1, 3 and 7 days after operation), ^#^ *P*<0.05 vs. TLR4-competent mice at the same time point.

**Fig 3:**
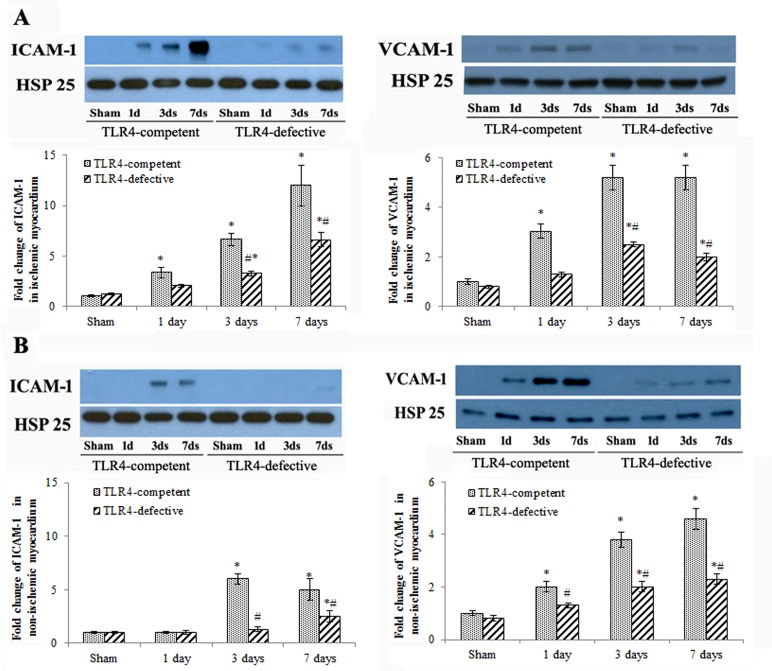
TLR4 deficiency reduces ICAM-1 and VCAM-1 levels in ischemic and non-ischemic myocardium. Representative immunoblots and densitometry data show that ICAM-1 and VCAM-1 levels are lower in ischemic (A) and non-ischemic (B) myocardium of TLR4-defective mice at day 1, 3 and 7 of reperfusion. Data are expressed as mean ± SE, n = 5 in each group, *P<0.05 vs. combined sham controls (1, 3 and 7 days after operation), ^#^ *P*<0.05 vs TLR4-competent mice at the same time point.

MCP-1 and KC levels in ischemic myocardium of TLR4-competent mice increased after 1 day of reperfusion and reached to peak levels after 3 days of reperfusion (Fig. 2A). The levels of MCP-1 and KC remained elevated at day 7 (Fig. 2A). ICAM-1 and VCAM-1 levels also increased in ischemic myocardium at day 1 to day 7 in TLR4-competent mice (Fig. 3A). TLR4-defective mice had lower levels of MCP-1, KC, ICAM-1 and VCAM-1 in ischemic myocardium at all time points (Figs. 2A and3A).

**Fig 4:**
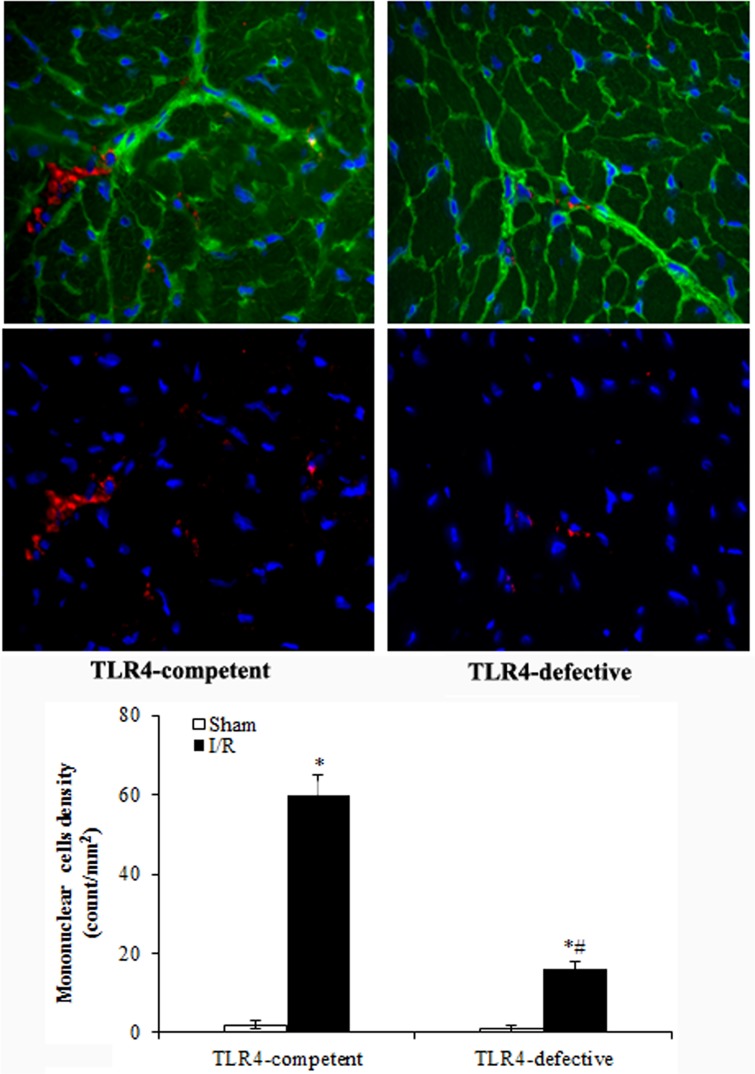
TLR4 deficiency reduces mononuclear cell accumulation in non-ischemic myocardium at day 7 of reperfusion. Mononuclear cells were labeled red using a specific antibody and counted with NIS-Elements basic research software. Representative immunofluorescence images and a bar graph show reduced mononuclear cell accumulation in non-ischemia myocardium of TLR4-defective mice. Data are expressed as mean ± SE. n = 5 in each group, *P<0.05 vs. sham controls, ^#^ *P*<0.05 vs. TLR4-competent mice.

Interestingly, the levels of MCP-1, KC, ICAM-1 and VCAM-1 also increased in the non-ischemic myocardium after I/R and exhibited similar temporal profiles as those in ischemic myocardium (Figs. 2B and3B). At day 3 of reperfusion, MCP-1 levels in non-ischemic myocardium of TLR4-competent hearts were 701 ± 62 pg/mg (*P*<0.05 vs. 260 ± 39 pg/mg in sham group), and KC levels in non-ischemic zone of TLR4-competent hearts were 230 ± 12 pg/mg (*P*<0.05 vs. 110 ± 10 pg/mg in sham group). TLR4 deficiency resulted in reduced levels of MCP-1 and KC in non-ischemic myocardium and abrogated the changes in ICAM-1 and VCAM-1 levels in non-ischemic myocardium (Figs. 2B and 3B). Similarly, mononuclear cell accumulation in non-ischemic myocardium was reduced in TLR4-defective mice (Fig. 4).

### TLR4-defective mice displayed reduced expression of MMP 9 and collagens in non-ischemic myocardium

We analyzed protein levels of MMP 2, MMP 9, TIMP-2 and TIMP-1, as well as MMP 9 and MMP 2 activity in the non-ischemic myocardium.

**Fig 5:**
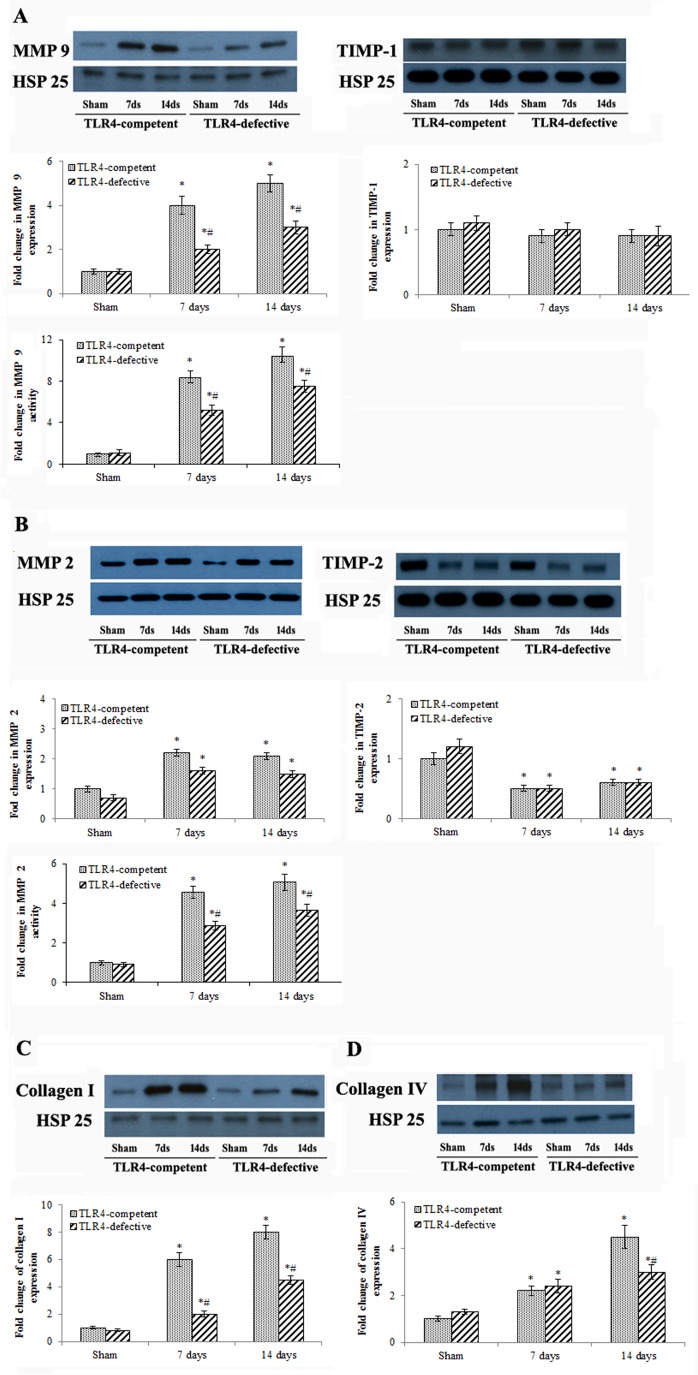
TLR4 deficiency reduces the expression of MMP 9, collagen I and collagen IV in non-ischemic myocardium at day 7 and 14. A & B. Representative immunoblots, blot densitometry data and results of enzyme activity assay show that protein levels and activities of MMP 9 (A), MMP 2 (B), are increased, but TIMP-2 protein levels (B) are decreased in non-ischemic myocardium of TLR4-competent mice at day 7 and 14 of reperfusion. TLR4-defective mice display lower levels of MMP 9 protein and activity, and lower MMP 2 activity in non-ischemic myocardium at day 7 and 14 of reperfusion. **C & D.** Representative immunoblots and densitometry data show that TLR4-defective mice have lower levels of collagen I (C) and collagen IV (D) in non-ischemic myocardium at day 7 and 14 of reperfusion. Data are expressed as mean ± SE. n = 5 in each group, *P<0.05 vs. combined sham controls (7 and 14 days after operation), ^#^ *P*<0.05 vs TLR4-competent mice at the same time point.

MMP 9 protein levels were significantly increased in the non-ischemic myocardium of TLR4-competent mice at day 7 (4 folds of sham control levels, *P*<0.05) and day 14 (5 folds of sham control levels, *P*<0.05) while TIMP-1 levels were essentially unchanged (Fig. 5A). In TLR4-defective mice, MMP 9 levels in non-ischemic myocardium were lower at day 7 and day 14 in comparison to those in TLR4-competent mice (Fig. 5A). MMP 2 levels were also increased in TLR4-competent mice at day 7 (2.2 folds of sham control levels, *P*<0.05) and day 14 (2.1 folds of sham control levels, *P*<0.05). In addition, TIMP-2 levels in non-ischemic myocardium were decreased at day 7 and day 14 in TLR4-competent mice (Fig. 5B). TLR4 deficiency had a moderate effect on the up-regulation of MMP 2 in non-ischemic myocardium, but had no effect on the down-regulation of TIMP-2 (Fig. 5B).

MMP 9 and MMP 2 activities were also elevated at day 7 and 14 of reperfusion in the remote no-ischemic myocardium of TLR4-competent mice (Fig. 5A and 5B). Compared to TLR4-competent mice, MMP 9 activity in TLR4-defective mice was 38% lower at day 7, and 28% lower at day 14 (Fig. 5A). Similarly, MMP 2 activity in TLR4-defective mice was 36% lower at day 7, and 27% lower at day 14 (Fig. 5B).

Both collagen I and collagen IV were increased in non-ischemic myocardium of TLR4-competent mice at day 7 and day 14 (Fig. 5C and 5D). At day 14, collagen I levels were 8 folds of sham control levels (*P*<0.05), and collagen IV levels were 5 folds of sham control levels (*P*<0.05). TLR4 deficiency markedly reduced collagen I and collagen IV levels in the non-ischemic myocardium at day 7 and day 14 (both *P*<0.05).

### TLR4-defective mice had improved LV function

**Fig 6:**
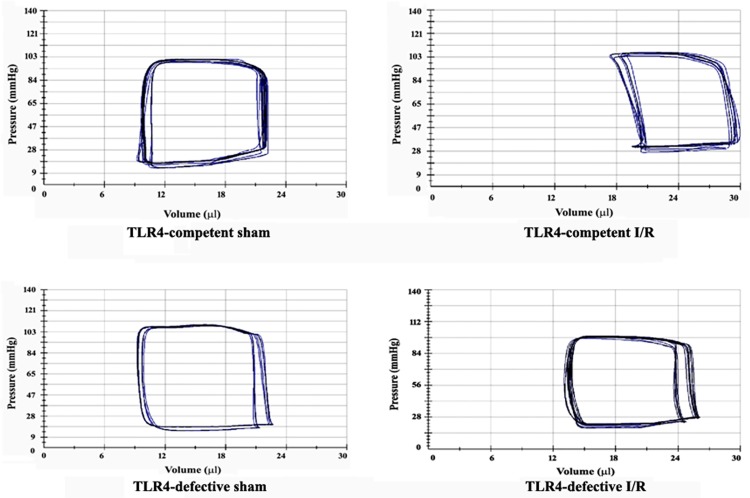
LV function is improved in TLR4-defective mice. LV function was analyzed by a mircrocatheter at day 14 of reperfusion. Representative pressure-volume loops show that TLR4-defective mice have improved LV functional performance following I/R compared to TLR4-competent mice.

We analyzed LV performance at day14 of reperfusion using a microcatheter. The LV functional parameters are listed in Table 1. Compared to TLR4-competent mice, LV developed pressure following I/R was markedly higher in TLR4-defective mice, and this LV functional parameter in TLR4-defective I/R hearts was not significantly different from that of sham control hearts. Ejection fraction was also markedly improved in TLR4-defective mice compared to TLR4-competent mice after I/R. Similarly, maximal elastance was higher in TLR4-defective mice than TLR4-competent mice. In TLR4-competent mice, significant increases in end-systolic volume and end-diastolic volume were observed following I/R, which caused a shift of the pressure-volume loop to the right (Fig. 6). In contrast, TLR4-defective mice had smaller end-systolic volume and end-diastolic volume. Meanwhile, elevated LV end-diastolic pressure was observed in TLR4-competent I/R hearts, but not in TLR4-defective I/R hearts (Fig. 6). Cardiac output was also improved in TLR4-defective mice after I/R (7.26 ± 1.11 ml/min vs. 5.30 ± 0.47 ml/min in TLR4-competent I/R; *P*<0.05).

**Table 1:** LV function parameters at day 14 after ischemia.

	TLR4-competent Sham	TLR4-competent I/R	TLR-defective Sham	TLR4-defective I/R
**Heart rates (bpm)**	621±62	570±45	633±73	585±51
**Developed pressure (mmHg)**	85.23±4.80	64.46±2.30*	88.86±10.50	83.14±5.90^#^
**End-systolic volume (**μ**l)**	9.03±1.22	33.09±1.59*	9.83±1.28	15.60±1.16* ^#^
**End-diastolic volume (**μ**l)**	22.03±1.53	42.98±2.36*	23.23±2.70	28.98±1.38* ^#^
**Cardiac output (ml/min)**	8.08±0.20	5.30±0.47*	8.21±1.09	7.26±1.11
**Ejection fraction (%)**	59.68±3.34	26.94±2.36*	59.82±8.31	46.53±3.21* ^#^
**Maximal elastance (mmHg/** μ**l)**	14.54±1.44	4.12±0.53*	14.57±2.07	9.4±1.28* ^#^

Mean ± SE; n = 7 in each group.

*P <0.05 vs. respective sham

^#^ *P*<0.05 vs. TLR4-competent IR

## Discussion

In the present study, we observed that the remote non-ischemic myocardium expresses chemokines and adhesion molecules from 1 to 7 days of reperfusion in a mouse model of regional myocardial I/R. The sustained inflammatory responses are associated with myocardial ECM protein remodeling at day 7 and day 14, and correlate with LV dysfunction at day 14. TLR4 deficiency markedly reduces these inflammatory responses in remote non-ischemic myocardium and attenuates ECM protein remodeling, resulting in a significant improvement in cardiac function.

### TLR4 plays an important role in regulation of the inflammatory responses of non-ischemic myocardium following regional I/R

Myocardial I/R elicits the inflammatory responses that involve complement activation, cytokine production and leukocyte infiltration [1]. A number of studies have found that TLR4 can sense the presence of a variety of endogenous molecules released from injured or stressed cells. Heat shock proteins, heparan sulfate, hyaluronan, biglycan, fibrinogen, high mobility group box-1 (HMGB-1) and several others have been implicated as endogenous activators of TLR4 signaling [7], [16–21]. It has been reported that hearts from TLR4-defctive mice and TLR4 knockout mice display attenuated NF-κB activation and reduced production of pro-inflammatory cytokine (TNF-α, IL-1β and IL-6) following myocardial ischemia with a short period of reperfusion [4], [16], [22]. These studies indicate that TLR4 plays an important role in mediating the myocardial inflammatory responses in the early phase of myocardial I/R injury.

Interleukin-8 (KC in murine) and MCP-1 are the key chemokines that regulate migration and infiltration of neutrophils and monocytes, respectively [23], [24], and ICAM-1 and VCAM-1 are critical molecules for leukocyte adhesion [25]. Our previous study found that myocardial TLR4 mediates the rapid up-regulation of MCP-1 and KC expression in isolated mouse hearts following global I/R [9]. In the present study, we found that levels of MCP-1, KC, ICAM-1 and VCAM-1 are increased at 1, 3 and 7 days after I/R not only in the ischemic area, but also in the remote non-ischemic myocardial tissue. Interestingly, the levels of these inflammatory mediators in non-ischemic myocardium are much lower in TLR4-defective mice in comparison to those in TLR4-competent mice. These results demonstrate that the remote non-ischemic myocardium also has sustained inflammatory responses to an I/R insult and that TLR4 is involved in mediating these responses.

It should be noted that MCP-1 and KC levels in non-ischemic myocardium are declining on day 7, and the significant difference between TLR4-competent and TLR4-defective mice is lost at this time point although the levels remain lower in TLR4-defective mice. It is likely that multiple mechanisms are involved in mediating the chemokine response and a certain mechanism, rather than TLR4, becomes dominant on day 7. Indeed, we observed that TLR4 deficiency reduces, but not abolishes, the chemokine response in the non-ischemic myocardium. In contrast, TLR4 deficiency results in the abrogation of the adhesion molecule response in non-ischemic myocardium at all of the time points examined. It seems that TLR4 plays a dominant role in mediating adhesion molecule expression in the non-ischemic myocardium at 1–7 days after I/R.

Mononuclear cell infiltration to the infarcted myocardium is required for the clearance of died cells and for injury repair through modulation of fibroblast activity and angiogenesis. In addition, infiltrated mononuclear cells modulate ECM remodeling [26–28]. MCP-1 is a critical factor for recruitment of mononuclear cells to injured tissue. In the present study, expression of MCP-1 in non-ischemic myocardium is associated with mononuclear cell accumulation in such tissue. Reduced MCP-1 levels in TLR4-defective mice correlate with reduced mononuclear cell accumulation in the non-ischemic myocardium. Mononuclear cells recruited into the infarct zone consist of two distinct phenotypes: Ly-6C^hi^ and Ly-6C^lo^. Ly-6C^hi^ cells dominate the early phase (phase I) of myocardial injury and exhibit phagocytic, proteolytic and inflammatory functions. Ly-6C^lo^ cells dominate late phase, have attenuated inflammatory properties, express vascular-endothelial growth factor and are involved in myocardial healing [29]. It appears that Ly-6C^hi^ cells can differentiate into Ly-6C^lo^ cells. While reduced myocardial MCP-1 production in TLR4-defective mice is appears to be responsible for attenuation of mononuclear cell accumulation in non-ischemic myocardium, it is not clear from the present study whether lack of TLR4 signaling affects the phenotypic transition of mononuclear cells in the injured areas.

### Decreased inflammatory responses in non-ischemic myocardium are associated with attenuated matrix protein remodeling

MMP 2 and MMP 9 are the components of ECM and have proteolytic functions. Their activities are regulated by natural specific inhibitors TIMP-2 and TIMP-1, respectively [30]. During the early stages of myocardial I/R injury, degradation of ECM proteins by MMPs results in progressive infarct expansion, ventricular wall thinning and ventricular dilation [31]. Clinical and animal studies showed that MMP, especially MMP 9, levels are elevated in myocardium after myocardial infarction, and an imbalance between specific MMPs and TIMPs occurs, contributing to LV dilation [32], [33]. It has been shown that selective inhibition MMP isoforms attenuates LV dilation after myocardial infarction [34]. In addition, TIMP-1 knockout mice have greater LV dilation and decreased ejection fraction after myocardial infarction, while inhibition of MMPs reverses ECM protein remodeling and compromised cardiac function [35].

In the present study, we found that both protein levels and activities of MMP 2 and MMP 9 are increased in the remote non-ischemic myocardium of TLR4-competent mice at day 7 and 14 after I/R. TIMP-2 levels are decreased at these two time points while TIMP-1 levels are unchanged. In addition, we found that collagen I and collagen IV levels are markedly increased in the remote non-ischemic myocardium of TLR4-competent mice at day 7 and day 14. Thus, I/R injury up-regulates MMP 2, MMP 9, collagen I and collagen IV and down-regulates TIMP-2 in non-ischemic myocardium. It appears that increased MMP 9 activity is primarily due the up-regulation of its protein levels, and both increased MMP 2 protein levels and decreased TIMP-2 protein levels account for the elevation of MMP 2 activity. TLR4 deficiency reduces both protein level and enzyme activity of MMP 9. While TLR4 deficiency has a moderate effect on MMP 2 protein level, it significantly reduces MMP 2 activity. Interestingly, the overall MMP 2/9 activity to protein ratio is slightly shifted in TLR4-defective mice compared to that of TLR4-competent mice although the cause remains to be determined. In various forms of human heart failure, increased MMP expression and activity, and a MMP/TIMP imbalance are closely related to collagen deposition and fibrosis in the myocardium [36]. In the present study, the reduced protein levels of MMP 2/9, as well as the altered ratio MMP 2/9 protein to activity in the remote non-ischemic myocardium of the TLR4-defective mice may lead to decreased collagen deposition or decreased ECM protein remodeling following I/R injury.

It is noteworthy that myocardial ECM protein remodeling in the non-ischemic myocardium of TLR4-competent mice is preceded by pro-inflammatory mediator production and mononuclear cell accumulation. It appears that TLR4-mediated inflammatory responses play an important role in up-regulation of the expression of MMP 9, collagen I and collagen IV caused by I/R. However, this innate immunoreceptor has a relatively minor role in mediating the changes in MMP 2 and TIMP-2 protein levels although it plays a role in elevating MMP 2 activity. We also observed that neutrophil infiltration in the ischemic myocardium is reduced at day 1 in TLR4-defective mice (not shown). Due to a transient nature of neutrophil infiltration and the absence of neutrophils in the remote non-ischemic myocardium (not shown), neutrophils may not have a significant effect on cardiac ECM protein remodeling observed ay day 7 and 14 of reperfusion.

Several studies on permanent regional ischemia models found elevated levels of MMPs [37] and collagens [13] in remote non-ischemic myocardium. However, the mechanism underlying ECM protein remodeling in non-ischemic myocardium remains unclear. A study by Timmers and colleagues [38] determined the effect of TLR4 mutation on ECM remodeling using a mouse model of permanent coronary ligation. Interestingly, ischemic myocardium, not the remote non-ischemic myocardium, displays elevated levels of MMP 2 and MMP 9 at day 4 in this model. Similarly, collagen density is increased at day 28 in the infarct and boarder zones, not in the remote non-ischemic myocardium. TLR4 deficiency does result in a reduction in MMP 2 and MMP 9 expression in the infarct zone in this study [38]. The marked difference between this study and our observation, as well as other reports, could be due to the differences in animal models utilized and the time course of observation. It should be noted, however, that long-term permanent myocardial ischemia is rare in humans. Many factors generated during early reperfusion are missing in an animal model of permanent myocardial ischemia, and these clinically relevant factors may elaborate the TLR4-mediated inflammatory responses and ECM protein remodeling in the remote non-ischemic myocardium.

Importantly, we observed in this I/R model that significantly lower protein levels of MMP 9, collagen I and collagen IV, as well as decreased MMP 9 and MMP 2 activity in the remote non-ischemic myocardium of TLR4-defective mice correlate to improved LV functional performance. Indeed, end-diastolic volume (a parameter of LV dilation) is reduced, and maximal elastance (a parameter of LV compliance) is increased at day 14 in TLR4-defective mice in comparison to TLR4-competent mice. Furthermore, ejection fraction and cardiac output are higher in TLR4-defective mice. Moderately reduced myocardial I/R injury (36% reduction in infarct size) and greatly suppressed ECM protein remodeling in the remote non-ischemic myocardium should contribute to the improvement of cardiac function in TLR4-defective mice. However, it remains unclear from the present study what inflammatory mediator causes ECM protein remodeling in the remote non-ischemic myocardium and which altered matrix protein is involved in the mechanism of cardiac dysfunction. These important issues need to be addressed in future studies.

## Conclusions

TLR4 signaling mediates the production of chemokines and adhesion molecules in remote non-ischemic myocardium following myocardial I/R. TLR4-mediated production of these pro-inflammatory mediators is followed by excessive production of MMP 9, collagen I and collagen IV, and increased MMP 9 and MMP 2 activity in the remote non-ischemic myocardium. The lack of TLR4 signaling reduces the expression of MMP 9, collagen I and collagen IV, and decreases MMP 9 and MMP 2 activity in the remote non-ischemic myocardium, and improves cardiac function following myocardial I/R. The results of the present study suggest that control of TLR4 activity or modulation of TLR4 signaling can suppress the inflammatory responses and ECM protein remodeling in the remote non-ischemic myocardium after myocardial I/R injury.
